# Effects of Cooling Rate on the Solidification and Microstructure of Nickel-Based Superalloy GTD222

**DOI:** 10.3390/ma12121920

**Published:** 2019-06-14

**Authors:** Bo Gao, Yanfei Sui, Hongwei Wang, Chunming Zou, Zunjie Wei, Rui Wang, Yanle Sun

**Affiliations:** 1 National Key Laboratory for Precision Hot Processing of Metals, School of Materials Science and Engineering, Harbin Institute of Technology, Harbin 150001, China; gaobolaile@126.com (B.G.); yanfei0620@sina.com (Y.S.); wanghw@hit.edu.cn (H.W.); zouchunming1977@163.com (C.Z.); weizj@hit.edu.cn (Z.W.); 2 Shanghai Key Laboratory of Advanced High-Temperature Materials and Precision Forming, School of Materials Science and Engineering, Shanghai Jiao Tong University, Shanghai 200240, China; 3 School of Materials Science and Engineering, Shanghai Jiao Tong University, Shanghai 200240, China

**Keywords:** GTD222, nickel-based superalloy, solidification behavior, cooling rate

## Abstract

In this work, the microstructure and solidification behavior of nickel-based superalloy GTD222 at different cooling rates are studied. The solidification of the superalloy GTD222 proceeds as follows: L → L + γ, L → L + γ + MC, L → L + (γ/γ′)-Eutectic and L → η phase. Due to alloying element redistribution, the temperature of the solidus GTD222 superalloy, 1310 °C, is slightly lower than the temperature of the liquidus, which is 1360 °C. It was found that the dendrite arm spacing of the alloy decreased with the increase of the cooling rate from 200 μm at 2.5 K/min to 100 μm at 20 K/min.

## 1. Introduction

Casting superalloys are widely applied in industrial areas such as gas turbines, aerospace and chemical process industries owing to their excellent mechanical properties and thermal corrosion resistance [1,2]. In order to develop more efficient advanced solidification technology, a data base of superalloys on thermophysical properties is increasingly needed [3]. From a theoretical and industrial perspective, knowledge of superalloy solidification behavior is crucial for the controlling of the superalloy casting process [4]. In the casting of nickel-based superalloys, the mechanical properties of alloys are due to microstructure characteristics, such as a combined matrix γ phase and γ′ precipitation phase [5], dendritic width [6] and grain size [7]. Hence, the optimum mechanical properties can be obtained only by applying suitable heat treatment [3]. 

For heat treatment after casting, the solidus temperature (the temperature at which incipient alloys begin to melt) and the formation temperature of precipitation (the onset precipitating of the γ′ phase, η phase and carbide phase), which determine the heat treatment window of materials, are very important. To obtain the ideal microstructure, heat treatment of the nickel-based superalloys must be carried out in certain temperature range. Therefore, for any nickel-based superalloy, solvus temperature and solidus temperature should be accurately measured, in order to optimize the mechanical properties of the superalloy. What is more, the incipient dissolving temperature of precipitation is another important parameter, helping to maximize the volume of precipitates without producing an interdendritic region.

In recent years, the demand of hot-end complex parts with different wall thicknesses has increased. The casting system of the hot-end parts is very intricate, and that leads to difficulty in controlling the microstructure. Numerous studies have shown a direct relationship between the cooling rate and material microstructure, such as the significant effect on the dendritic width by the cooling rate of solidification process. Chen et al. [8] studied the compositional changes of the micro-scale precipitates of an advanced Ni-base superalloy at different cooling rates. It was found that the chemical composition of the precipitates of different sizes is very different. This study has important implications for understanding the microstructure and precipitation behavior of Ni-based superalloys. Zheng et al. have testified that the cooling rate significantly influences the morphology of dendrites [9]. The dendrite arm spacing of both primary and secondary dendrite declined as the cooling rate increased [10,11,12]. GTD222, a nickel-based precipitation hardened isometric crystal superalloy, is considered to be one of the most suitable superalloys that can be processed into the guide vane of a steam turbine, servicing at 1000 °C [13]. Most work focused on the optimization of the composition of the GTD222 superalloy. However, less attention has focused on the solidification behavior of the GTD222 superalloy [14]. In this study, an empirical research was carried out to understand the effect of cooling rate on the solidification behavior and microstructural evolution of the GTD222 superalloy, and the liquidus temperature and solidus temperature of the GTD222 superalloy were also measured.

## 2. Experimental Procedures

The chemical composition of the GTD222 superalloy used in the present work is given in Table 1. Commercial pure metals (> 99.95 wt.%) were used for the preparation of the alloy prior to melting. To ensure compositional homogeneity, the alloy melt was fully stirred by an electromagnetic stirring system equipped in the arc furnace (QSH-ZP, Quanshuo, Shanghai, China), and each button alloy was flipped and melted at least four times. The materials were firstly prepared in a vacuum induction melting furnace, and then casted into ingots (100 mm × 100 mm × 150 mm). All specimens used in this work were cut from the ingot using a spark cutting machine (Dk77, Zhonggu, Suzhou, China).

The solidification procedure of the GTD222 superalloy was revealed by differential scanning calorimetry (DSC, DSC 404 F3 Pegasus, NETCH, Selb, Germany). All DSC testes were carried out by alumina crucibles in the argon protection environment with sample sizes of 2.5 mm in diameter and 2 mm in height. The cooling rates of samples were 2.5 K/min, 5 K/min, 10 K/min and 20 K/min, respectively.

Microstructural evolution and phase constitutions of GTD222 samples were carried out on an optical microscope (OM, Axio Imager A1m, ZEISS, Jena, Germany), X-raydiffraction (XRD, XRD-6000 diffraction instrument, Shimadzu, Kyoto, Japan), X-ray energy-dispersive spectroscopy (EDS, JSM-7600F, Tyoto, Japan) and scanning electronmicroscope (SEM, Sirion 200, FEI, Hillsboro, OR, USA). The samples for OM and SEM were ground to 2000 grit, and then polished by the diamond polishing paste (1 μm). The etchant for samples was 45 mL CuSO_4_, 100 mL H_2_O and 50 HCl. Phase constitutions of the alloy were determined by X-ray diffraction (XRD) technique, using Cu Kα (λ = 0.1540562 nm) radiation, operating at 40 kV and 30 mA between 20° and 80° (2θ) at a step of 0.02° and a counting time of 0.6 s per step.

## 3. Results and Discussion

### 3.1. Microstructure of As-Cast Alloy

**Figure 1:**
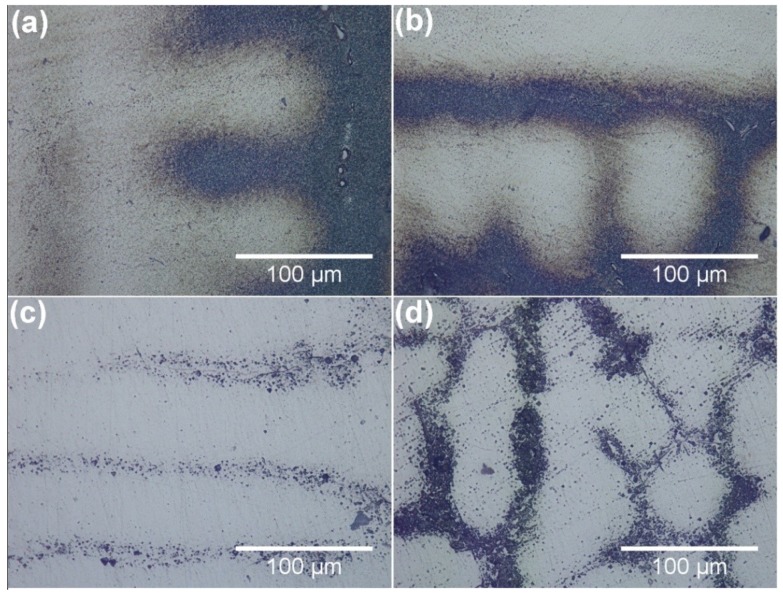
Optical microscope (OM) images of the as-cast GTD222 superalloy with different cooling rates during the solification process, (**a**) cooling at 2.5 K/min, (**b**) cooling at 5 K/min, (**c**) cooling at 10 K/min, (**d**) cooling at 20 K/min.

**Figure 2:**
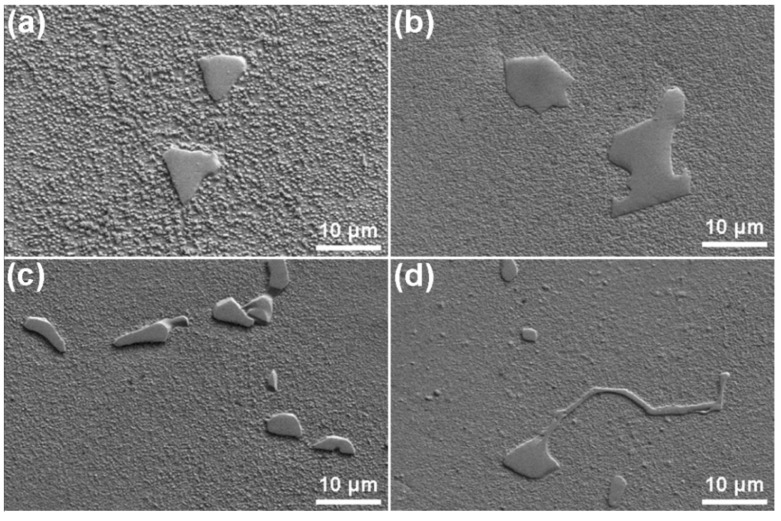
SEM images of the as-cast GTD222 superalloy with different cooling rates during the solification process, (**a**) cooling at 2.5 K/min, (**b**) cooling at 5 K/min, (**c**) cooling at 10 K/min, (**d**) cooling at 20 K/min.

Figure 1 and Figure 2 show the microstructure of an as-cast GTD222 superalloy with different cooling rates. The microstructure of the GTD222 superalloy is a typical dendritic structure in the as-casting samples, and no equiaxed grains were found in Figure 1. The dendritic structures in Figure 1a are coarser than dendritic structures in Figure 1b–d. It can be seen that the dendritic structures are coarsening, along with the decrease in the cooling rate. Additionally, the secondary dendritic arm spacing has the same tendency. According to V. Kavoosi, the secondary dendritic arm spacing is related to the local cooling rate [15]. The size of the secondary dendrites directly affects the composition segregation, the second phase and the distribution of micropores. As shown in Figure 2, the size of the carbide precipitation is decreasing as the cooling rate increases from 2.5 K/min to 20 K/min, since a low cooling rate would provide more time for the diffusion of the atoms and coarsen the second phase. Furthermore, the secondary dendrite arm spacing of the alloy is closely related to tensile strength and elongation. Generally, the smaller the distance between secondary dendritic arms, the better the mechanical properties produced. The high cooling rate would refine the grain and dendrite arm spacing of materials, producing a greater fraction of boundaries and consequently improving the mechanical performance of the materials at ambient temperature. However, the boundaries would become weak phase in the elevated temperature atmosphere [16,17]. More grain boundaries and finer dendritic arm spacing would make the mechanical properties of the materials decrease dramatically. Therefore, it is important to find a suitable cooling rate to optimize the potential of the material to control the microstructure of the material and thus to optimize the properties of the superalloy.

### 3.2. Solidification Process

**Figure 3:**
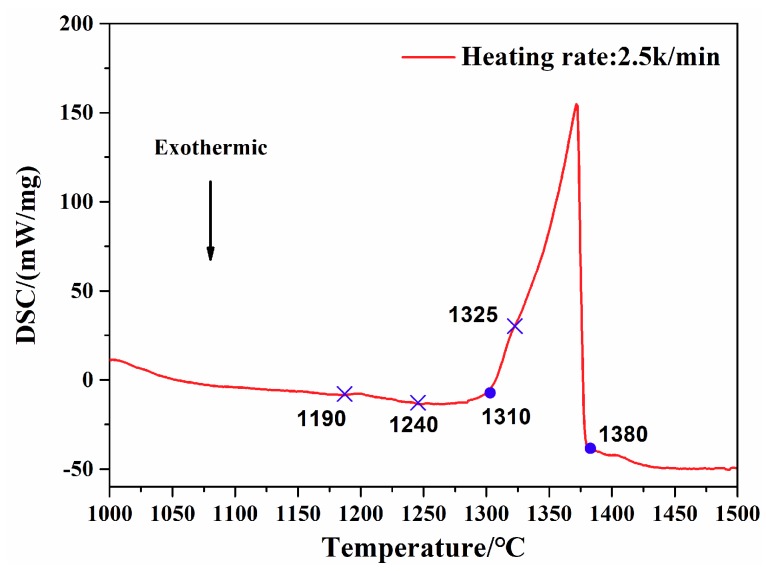
Heating differential scanning calorimetry (DSC) curve of the as-cast GTD222 superalloy with a heating rate of 2.5 K/min.

Figure 3 shows the heating DSC curves of the GTD222 superalloy sample cutting from the as-casting ingot, the heating rate of the sample is 2.5 K/min. The solidus temperature of the matrix γ phase can be identified from the heating curve. The value of γ phase solidus temperature is 1310 °C. The ending temperature of the endothermic peak corresponding to the matrix γ phase is 1380 °C. According to the DSC curve, the beginning dissolution temperature of the γ′ phase is 1190 °C. The temperatures of 1240 °C and 1325 °C represent the ending solution temperature of γ′ phase and the beginning solution temperature of the carbides, respectively. It is worth noting that it is difficult to retrieve the dissolving values of γ′ phase from the measurements of the DSC heating curve, owing to the noisy background accompanied with sluggish heating rate of 2.5 K/min [3]. 

**Figure 4:**
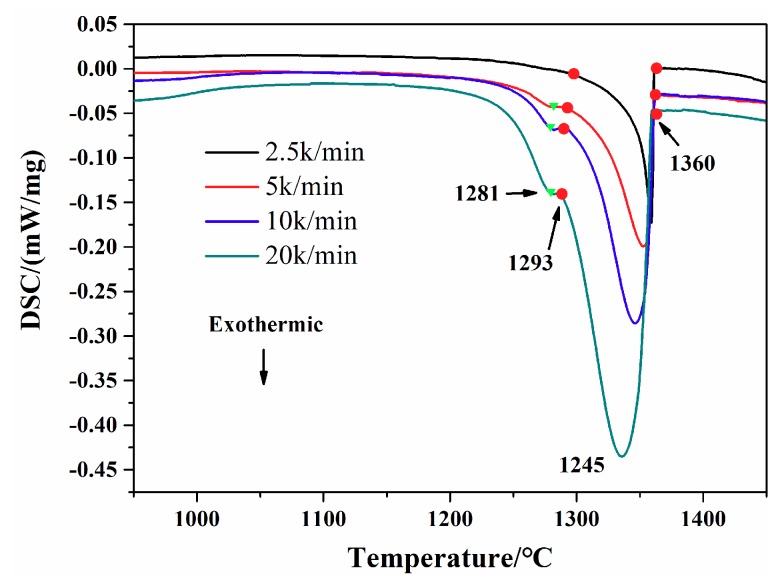
DSC curves of the alloy cooled from super-solidus temperature, the cooling rates are 2.5 K/min, 5 K/min, 10 K/min and 20 K/min, respectively.

Figure 4 shows the DSC curves of samples with a cooling rate of 2.5 K/min, 5 K/min, 10 K/min and 20 K/min, respectively. All the DSC samples were heated to 1500 °C with a heating rate of 2.5 K/min and held for 10 min at this temperature prior to the cooling process down to 700 °C. Taking the curve of the sample with a cooling rate of 20 K/min as an example, two obviously exothermic peaks were observed as the sample cooled down to 700 °C. From high temperature to low temperature, the first and largest exothermic peak is related to the process of solidification of the matrix γ phase, which can be described as the following formula: L-L + γ. The initiating temperature of this exothermic peak of the cooling DSC curve is 1360 °C, closer to the other three curves with different cooling rates. The end temperature of this exothermic peak is about 1293 °C, slightly lower than the other curves. It can be seen from the curves that the ending temperature of the first peak decreased as the cooling rate increasing from 2.5 K/min to 20 K/min. Followed by the first exothermic peak, the second exothermic peak with an ending temperature of 1281 °C was observed. The heating curve was used to obtain the solidus, and the cooling curve was used to quote the liquidus [3]. Therefore, from the curves shown in Figure 3 and Figure 4, the solidus of the GTD222 superalloy can be determined as 1310 °C and the liquidus can be determined as 1360 °C. Figure 4 also shows that the exothermic peak of matrix γ phase became sharper when the cooling rate increased from 2.5 K/min to 20 K/min, with a slight decline of the non-equilibrium phase transition temperature. Since undercooling of materials increased along with the increasing of cooling rate, the enhanced undercooling of the sample with a high cooling rate would rapidly release large latent heat, leading to the recalescence effect that remelted the solidified primary γ phase. The partial re-melted matrix γ phase would cause solidification and release the heat again [18]. That is why the exothermic peak of γ phase is becoming sharper and the ending temperature of the first peak becoming lower as the cooling rate increases. A large cooling rate from the molten state would produce an enhanced undercooling, increase the driving force of the formation of non-equilibrium phase, and promote the precipitation of the non-equilibrium phase. For the competition between the non-equilibrium phase and the primary γ phase, dendrites at larger cooling rates are finer than the dendrites obtained at small cooling rates, as shown in Figure 1.

**Figure 5:**
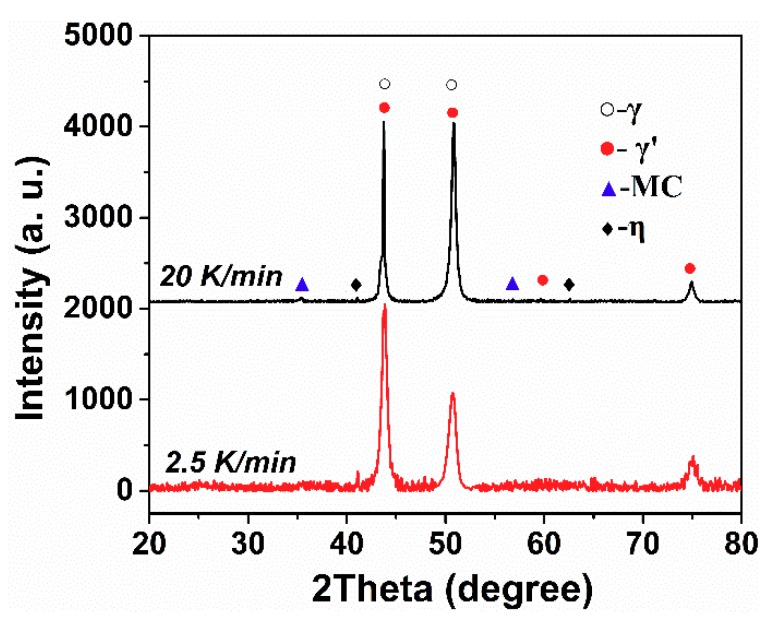
The XRD patterns of the GTD222 superalloy with a cooling rate of 2.5 K/min and 20 K/min, respectively.

**Figure 6:**
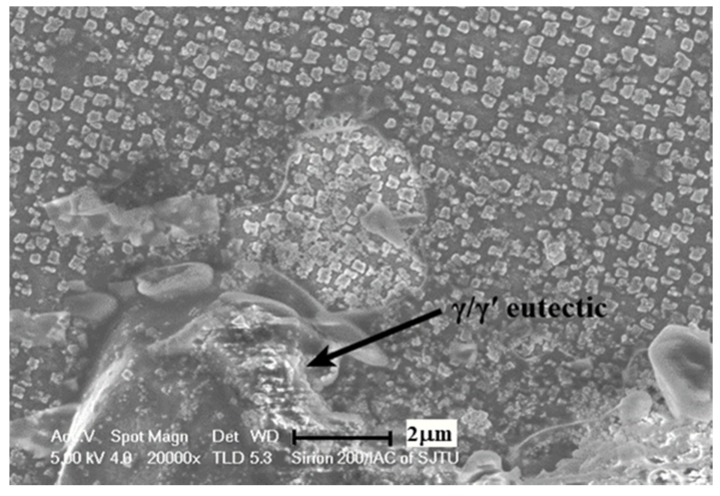
Petal γ/γ′ eutectic phases in the interdendritic area of the DSC sample with a cooling rate of 20 K/min.

**Figure 7:**
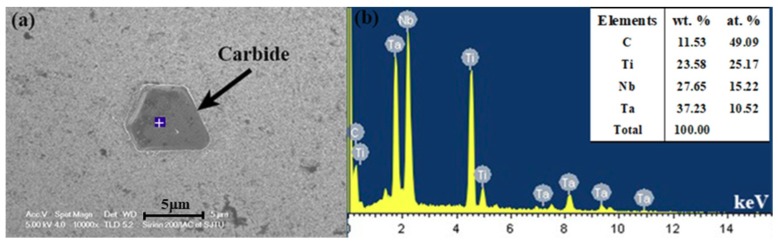
Carbide phase in the solidified DSC sample with cooling rate of 20 K/min. (**a**) is the SEM morphology of carbide. (**b**) is the EDS spectrum of the spot of carbide in the DSC sample, according to the white cross in blue background. The inset in top right corner is the component ratio in weight and atom.

**Figure 8:**
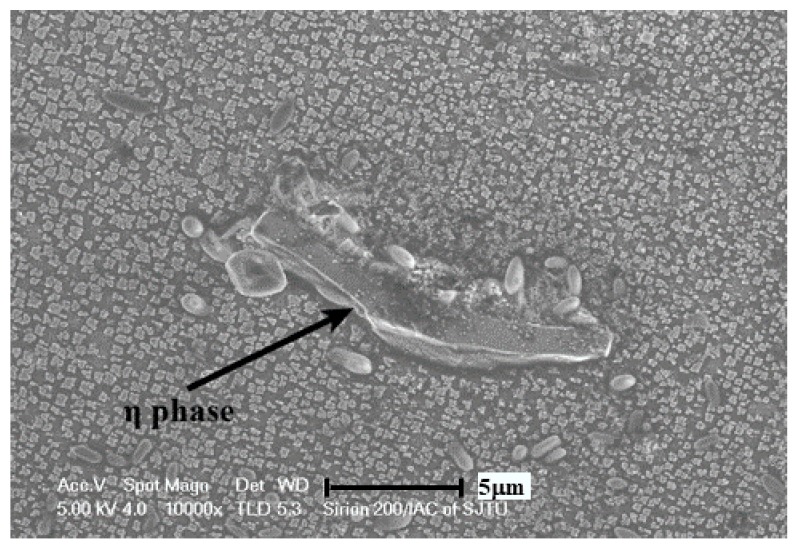
η phase in the interdendritic area of the solidified DSC samples with cooling rate of 20 K/min.

On account of the complexity of the phases in the GTD222 superalloy, it is arbitrary to identify what the exothermic peak is to refer to the curve itself. Therefore, the phase analysis, and accordingly the solidification process of the GTD222 superalloy, was carried out using XRD. Figure 5 is the XRD pattern of the GTD222 superalloy obtained with a cooling rate of 2.5 K/min and 20 K/min, respectively. Figure 5 shows the phases of the GTD222 superalloy, including γ′ phase, MC phase and η phase. Figure 6, Figure 7 and Figure 8 show different phases observed in the interdendritic areas of the samples. Figure 6 is a typical morphology of the γ + γ′ eutectic phase of the GTD222 superalloy that was densely distributed in the interdendritic. Figure 7 shows the carbide phase precipitation. As shown in Figure 7b, the carbide phase precipitation is rich in Ti, Nb and Ta. Figure 8 is the typical morphology of the η phase with a coarse needle-like configuration reported in cast Ni-based alloy [19,20]. Note that only the γ′ phase can be observed in Figure 6, since the γ phase was too fragile to resist the corrodent and disappeared during detecting. The morphology of the γ′ phase also varied when the Ti/Al atom ratio changed [21]. The γ′ phase in γ/γ′ eutectic phase is needle like while the primary γ′ phase is cube like in the present work. 

## 4. Discussion

### 4.1. Formation of the γ/γ′ Eutectic Phases

The γ/γ′ eutectic phase is not thermodynamic equilibrium, and the emergence of this phase can be attributed to the solution segregation of elements that occurrs during the solidification of the GTD222 superalloy [22]. The segregation behavior of the elements in alloys is inevitable, since there are differences in the diffusion rates and melting points of different elements. During the solidification of the GTD222 superalloy, the Al is rejected into the liquid phase continuously. At last, the content of Al with a lower melting point is rich enough to form the γ/γ′ eutectic phase. The formation process of γ/γ′ eutectic phase of GTD222 could be depicted as following: the first emerging phase γ is precipitated in the liquid phase while the Al in the liquid is rejected into the residual liquid phase; with the increase of Al content the γ/γ′ eutectic phase, Ti is continuously rejected into the liquid phase. The formation of the γ/γ′ eutectic phases and γ′ phase during the solidification of the GTD222 superalloy should occur almost simultaneously, and it is hard to separate the exothermic peaks related to those two phases completely.

The third phase found in the as-cast samples of the GTD222 superalloy is carbide precipitation. Figure 7a is the typical morphology of the carbide precipitation phase, which can be identified by EDS, giving the elements the composition that was shown in Figure 7b. The elements composition in Figure 7b is typical of the components of carbide, which is rich in Ti, Nb and Ta elements. The molar ratio of the metal element (the sum of Ti, Nb and Ta) to the carbon element is about 1:1. Based on the outcomes of EDS, the carbide can be the same as the MC-type carbide [23]. It is hard to find out the exothermic peak of carbide on the cooling curve of DSC but the endothermic peak of carbide on the heating curve of DSC can be easily confirmed.

### 4.2. Formation of the η Phase

Some researchers have confirmed that the ultimate formation phase of nickel-based superalloy with B element is the boride phase, which is rich in Mo and Cr elements [24]. However, in the present study no Mo or Cr rich phase was found and no exothermic peak corresponding to boride was found on the DTA curve of the GTD222 superalloy. The distribution mapping of the B element is hard to trace owning to its low content. In the present superalloy, the formation of η phase represents the ending of the solidification process of the GTD222 superalloy. During the subsequent cooling, the γ′ precipitates start to precipitate from the matrix γ phase, which corresponds to the third inconspicuous exothermic peak on the cooling DSC curve. The onset temperature of the third exothermic peak is hard to determine. This is due to the difference in elements solute distribution between dendrite area and interdendritic area. As a result, the precipitation temperature of the γ′ phase is different in different areas. The stack of the exothermic heat of all the γ′ phases in the different regions forms the third exothermic peak on the cooling DSC curve and that is why the third exothermic peak temperature range is so large.

Based on the discussion above, the formation sequence of phase during the solidification of the GTD222 superalloy can be summarized as follows: liquid translates into liquid and the γ phase, liquid translates into liquid and the carbide phase, liquid translates into liquid and the γ/γ′ eutectic phase, liquid translates into the η phase. The onset temperatures of precipitation for all these phases vary when the cooling rate of alloy is changed. When the cooling rate is 20 K/min, the onset temperature of the second exothermic peak is 1293 °C. Compared with a sample cooling at 10 K/min, it can be observed that the solidus of a 20 K/min sample is slightly lower than the solidus of a sample cooling at 10 K/min. The phase transformation of the GTD222 superalloy solidification behavior under different cooling rates, the solidus temperature, and the liquidus temperature are important components of GTD222 superalloy thermophysical data. Those thermophysical data are the basis for designing the casting process of the alloy and exploring the potential capacity of the superalloy by using heat treatment. 

## 5. Conclusions

1. The solidification of the GTD222 superalloy proceeds as follows: L → L + γ, L → L + γ + MC, L → L + (γ/γ′)-Eutectic and L → η phase. The type of carbide precipitation is MC-type carbide in terms of the component ratio in atom, according to EDS analysis. Then, the formation of the γ/γ′ eutectic phases and the γ′ phase simultaneously occurred during the solidification. As the solidification proceeds, the formation of the η phase represents the ending process of the GTD222 superalloy solidification. Owing to the alloying elements redistribution in the solidification process, the temperature of the solidus of the GTD222 superalloy, 1310 °C, is slightly lower than the liquidus temperature, which is 1360 °C.
2. As the cooling rate increased from 2.5 K/min to 20 K/min, the dendrite arm spacing decreased from 200 μm at 2.5 K/min to 100 μm at 20 K/min. Also, the size of the precipitation phase decreased as the cooling rate increased. According to these three precipitation phases, the onset temperature of precipitation phases varied as the cooling rate of the alloy changed.

## Figures and Tables

**Table 1:** Chemistry (wt.%) of the GTD222 superalloy.

C	Cr	Co	W	Al	Ti	Nb	B	Ta	Ni
0.08–0.12	22.2–22.8	18.5–19.5	1.8–2.2	1.0–1.4	2.1–2.5	0.7–0.9	0.002–0.007	0.9–1.1	Bal.
